# A Heart and A Mind: Self-distancing Facilitates the Association Between Heart Rate Variability, and Wise Reasoning

**DOI:** 10.3389/fnbeh.2016.00068

**Published:** 2016-04-08

**Authors:** Igor Grossmann, Baljinder K. Sahdra, Joseph Ciarrochi

**Affiliations:** ^1^Department of Psychology, University of WaterlooWaterloo, ON, Canada; ^2^Institute for Positive Psychology and Education, Faculty of Health Sciences, Australian Catholic UniversityStrathfield, NSW, Australia

**Keywords:** heart-rate variability, psychological distance, reasoning, wisdom, attributions, vagal tone, egocentrism

## Abstract

Cardiac vagal tone (indexed via resting heart rate variability [HRV]) has been previously associated with superior executive functioning. Is HRV related to wiser reasoning and less biased judgments? Here we hypothesize that this will be the case when adopting a self-distanced (as opposed to a self-immersed) perspective, with self-distancing enabling individuals with higher HRV to overcome bias-promoting egocentric impulses and to reason wisely. However, higher HRV may not be associated with greater wisdom when adopting a self-immersed perspective. Participants were randomly assigned to reflect on societal issues from a self-distanced- or self-immersed perspective, with responses coded for reasoning quality. In a separate task, participants read about and evaluated a person performing morally ambiguous actions, with responses coded for dispositional vs. situational attributions. We simultaneously assessed resting cardiac recordings, obtaining six HRV indicators. As hypothesized, in the self-distanced condition, each HRV indicator was positively related to prevalence of wisdom-related reasoning (e.g., prevalence of recognition of limits of one’s knowledge, recognition that the world is in flux/change, consideration of others’ opinions and search for an integration of these opinions) and to balanced vs. biased attributions (recognition of situational and dispositional factors vs. focus on dispositional factors alone). In contrast, there was no relationship between these variables in the self-immersed condition. We discuss implications for research on psychophysiology, cognition, and wisdom.

## Introduction

Though wisdom can be viewed in different ways (Staudinger and Glück, 2011), many social scientists view it through a cognitive lens, as an unbiased judgment informed by knowledge of the social world (Assmann, 1994; Baltes and Staudinger, 2000; Baltes and Smith, 2008; Grossmann et al., 2010). In parallel, behavioral neuroscientists have suggested that wisdom involves the heart, or visceral functions (Cannon, 1932; Damasio, 1994; Meeks and Jeste, 2009). Cardiac vagal tone, indexed via heart rate variability (HRV), is one prominent marker of such visceral functioning. It has been linked to higher executive functions (Thayer et al., 2009) and emotion-regulation (e.g., Fabes and Eisenberg, 1997). The present report bridges these perspectives, examining whether and how HRV functioning is related to indices of a wisdom-related judgment. Drawing on the prior literature, we propose that the link between HRV and wisdom-related judgment may not be straightforward. Rather, we propose that higher-HRV individuals may exhibit wiser judgment when they are encouraged to transcend their egocentric impulses via self-distancing instructions.

### Heart Rate Variability

Human heart rate tends to fluctuate, even during steady-state conditions, such as while a person is resting. HRV refers to the variation in the time interval between heartbeats, which occurs through the actions of the sympathetic and parasympathetic branches of the autonomic nervous system. Parasympathetic influence on heart rate often acts to dampen sympathetic influence via innervation of the vagus nerve, which acts as a brake on the activity of the heart’s natural pacemaker, the sympathetically driven sinoatrial node. HRV indicators may represent, in part, the related phenomenon of respiratory sinus arrhythmia—the periodic variation in heart rate that occurs during a breathing cycle and which is linked to the functioning of the vagus nerve. People with higher resting HRV have shown greater activity in neocortical structures implicated in executive functioning such as the anterior executive region (Thayer and Brosschot, 2005; Thayer and Lane, 2009; Thayer et al., 2012). They also show superior performance on tasks measuring executive functioning, including working memory and go/no-go tasks (Hansen et al., 2003; Thayer et al., 2009), and on tasks measuring flexible and adaptive emotional responding (Ruiz-Padial et al., 2003; Appelhans and Luecken, 2006; Thayer et al., 2009) and self-control (Segerstrom et al., 2011). In contrast, people with lower resting HRV show hypoactive subcortical structures (Ahern et al., 2001), suggesting poorer cognitive and emotional functioning, and reduced wellbeing, as indicated by morbidity, mortality, or risk of heart disease and the stroke (Thayer and Lane, 2007).

To account for these findings, Thayer and colleagues proposed the neurovisceral integration model (Thayer and Lane, 2000; Thayer et al., 2012). This model suggests that resting HRV is tied to the functioning of prefrontal-subcortical circuits, such that higher resting HRV is associated with the effective functioning of prefrontal-subcortical executive circuits that support flexible and adaptive responses to environmental demands (Thayer and Lane, 2000; Thayer et al., 2009). In this view, HRV provides an index of how strongly “top-down” appraisals, mediated by cortical-subcortical pathways, shape autonomic responses of the body (Thayer et al., 2012; Park and Thayer, 2014). Consistent with this model, higher HRV may be conceptualized as having an effective “vagal brake,” wherein there is a flexible redirecting of energy from the periphery to the brain during regulation of mental efforts (e.g., Thayer et al., 2009; Bertsch et al., 2012), making glucose available for the metabolic costs of social information processing (Porges, 2001).

### Heart Rate Variability and Wisdom

On the surface, the centrality of HRV-mediated cortical executive circuits for efficient processing of a problem or situational demands suggests that people with higher HRV would reason in a wiser fashion. However, philosophers and psychological scientists suggest that problem-solving efficiency and generalized cognitive abilities are insufficient for a wiser judgment (Sternberg, 1998; Staudinger and Glück, 2011; Grossmann et al., 2013). For example, people may use their cognitive skills to make “unwise” decisions, such as those that fail to take into account other people’s view point and produce discrimination. Given the heterogeneous nature of the wisdom construct (Staudinger and Glück, 2011), in Sections “Wisdom as an Unbiased Social Judgment” and “HRV and Egocentrism” below we consider several wisdom-related processes and their relationship to HRV.

#### Wisdom as an Unbiased Social Judgment

A central lay notion of wisdom is close to the characterization of the biblical King Solomon, who is often portrayed for his *unbiased* judgment about matters of social life (Assmann, 1994). Consider a decision involving evaluation of another person’s behavior. Is this behavior due to the person’s dispositions or is it also influenced by the situation? In societies emphasizing the centrality of the self (Markus and Kitayama, 1991), people tend to be biased towards dispositional over situational explanations, even when situational explanations are correct (cf. fundamental attribution error [FAE]; Ross, 1977). Conversely, a wiser, less biased judgment would involve consideration of both situational and dispositional factors. People also tend to have an egocentric tendency to generate evidence in support of their initial views, discounting others’ perspectives (cf. myside bias; Greenhoot et al., 2004; Johnson-Laird, 2006). In both examples, self-centrality is the default response and inhibits an unbiased, wiser judgment.

A growing consensus suggests that wisdom-related *cognitive processes* also involve a set of pragmatic reasoning strategies that helps people navigate challenges of social life (e.g., conflicts between groups and individuals; Grossmann et al., 2010, 2013; also see Staudinger and Glück, 2011). Aspects of such reasoning include recognizing the limits of one’s knowledge, being aware of the varied contexts of life and how they may unfold over time, acknowledge other people’s points of view, and search for reconciliation of opposing viewpoints (Baltes and Smith, 2008; Grossmann et al., 2013). These processes can be captured by evaluating participants’ responses when reflecting on various societal or interpersonal matters (cf. Grossmann et al., 2010, 2012, 2013). Similar to the role of self-centrality for biased social judgments, a common feature underlying these different facets of wise reasoning is that they require people to transcend their egocentric impulses (Staudinger and Glück, 2011; Kross and Grossmann, 2012; Grossmann and Kross, 2014).

#### HRV and Egocentrism

Some studies indicate that wise reasoning is not directly related to HRV-mediated executive functioning among healthy adults (Grossmann et al., 2013). Similarly, greater cognitive abilities and executive functioning do not necessarily prevent egocentrism and inductive reasoning biases that result from it (Sternberg, 1998; Stanovich and West, 2007, 2008). Indeed, there is evidence that in some contexts, the more people think about an issue, the more biased they become (Petty et al., 2009). Together, these observations suggest that the relationship between HRV and wise reasoning may not be straightforward and depend on person’s tendency to transcend egocentric impulses.

People vary in their egocentric tendencies (Foster et al., 2003). When processing information in an egocentric fashion, high-HRV people may be very efficient. However, egocentric high-HRV people may choose to attend accurately to the self-serving features of the social issue at hand, discounting the non-self-serving features, including interests and opinions of other individuals involved in the issue. Similarly, high-HRV people who are particularly prone to egocentrism may generate a multitude of reasons for one’s favored opinion, resulting in a greater likelihood of discounting likely, but unfavorable views.

To our knowledge, only a handful of studies have explored the relationship between egocentric (vs.-prosocial) tendencies and trait-HRV directly, producing inconclusive results. In one of these studies, researchers observed that people with moderate trait-HRV are perceived as more “trustworthy,” “compassionate,” “and “kind” than people with low or high trait-HRV (Kogan et al., 2014). It is possible that these impressions reflected person’s superior impression management abilities rather than reduced egocentrism. In contrast, another study reports a positive linear relationship between HRV and a marker of prosociality concerning social connectedness (Kok and Fredrickson, 2010). Greater trait-HRV has also been associated with successful cultural socialization in the USA (Beauchaine, 2001), including cultivation of positive emotions (Eisenberg et al., 1995; Fabes and Eisenberg, 1997; Oveis et al., 2009), focus on which is more normative in the USA than elsewhere (Tsai, 2007; Grossmann and Kross, 2010; Grossmann et al., 2012, 2014, 2015; Grossmann and Na, 2014; Koopmann-Holm and Tsai, 2014; Ford et al., 2015). Yet, greater sensitivity to cultural norms (cf. Grossmann and Na, 2014) does not need to translate into less egocentrism.

### Self-Distancing and HRV

Kok and Fredrickson (2010) observed that trait-HRV was positively related to social connectedness, but only when instructing participants to engage in loving-kindness-meditation. This finding is noteworthy, because this meditation typically starts with kindness towards oneself and progresses to kindness towards loved ones, then strangers, and then the entire humanity, orienting people beyond self-centered interests. This finding also dovetails with a body of research dealing with curbing people’s egocentric impulses: Instructing participants to adopt a self-distanced perspective (visualizing the experience from an observer’s perspective), as compared to the baseline self-immersed perspective (visualizing the experience through their own eyes; e.g., Kross et al., 2005, 2012; Ayduk and Kross, 2010; Grossmann and Kross, 2010; Kross and Ayduk, 2011; Mischkowski et al., 2012). Research indicates that cueing people to reflect on social experiences from a self-distanced perspective leads them to focus less on egocentric recounting of their experiences and more on reconstruing the experience in ego-decentered ways that facilitate insight and reconciliation of disagreements, helping them to work through social dilemma they encounter in their lives (Kross et al., 2005; Gruber et al., 2009).

Based on these findings and past theory (Thayer and Lane, 2000; Thayer et al., 2012), we propose that the key factor distinguishing conditions under which higher HRV would be linked to a wiser judgment concerns the role of egocentrism and its attenuation through a self-distanced perspective (as compared to a self-immersed perspective). Specifically, we propose that high HRV people will be abler than low HRV people to reflect on a social issue in a wise fashion when they are cued to self-distance. However, when they are not cued to self-distance (self-immersed), both low and high HRV people are expected to show the default egocentric biases.

### Study Overview

We assessed behavioral processes reflecting wisdom-related judgment in two ways. First, we asked participants to reason about societal issues and examined their narratives for multiple aspects of wisdom-related reasoning strategies. Second, we asked participants to reflect on desirable/undesirable acts committed by another person and tested whether their judgments of those acts relied on biased dispositional explanations or more balanced situation-sensitive explanations. Throughout the study, we measured resting electrophysiological signature of the heart—the HRV, obtaining a range of time- and frequency-domain HRV indicators. To examine how self-distancing moderates the relationship between wisdom-related judgment and HRV, participants were randomly assigned to adopt a self-distanced as compared to the self-immersed perspective when reflecting on the social issue.

## Materials and Methods

Assuming a correlation of 0.21, which corresponds to the average effect size in social psychology and personality research (Richard et al., 2003), power analysis suggested a sample size of 176 for an alpha = 0.05 and beta = 0.20 (power = 0.80). We oversampled 10 participants to account for exclusion due to medical conditions. We recruited 186 participants at the University of Western Sydney, NSW, Australia. We excluded participants who did not follow instructions (2.2%), who stated they did not care about societal issues (2.7%), or whose audio-recording was missing (1.6%). We also excluded participants who had cardiac/neurological illnesses, who were taking heart-functioning related medicine (e.g., beta-blockers), or whose respiration rate was outside the range of 0.12–0.40 Hz within which HF-HRV is interpretable (13.4%), because these are all confounding factors for HRV analyses (Berntson et al., 1997). The final sample consisted of 150 adults (74.7% students, *M*_age_ = 25.48, *SD*_age_ = 9.22, female = 64.7%, male = 35.3%).

### Procedure

All participants provided written informed consent. The study had full ethics clearance from the University of Western Sydney’s Human Research Ethics Committee (H9798). The experimenter greeted participants and obtained informed consent, at which time participants were accompanied to a computer terminal, where they were connected to the ECG equipment. Orthostatic resting 6 min recordings (ECG Lead II configuration) were taken at the beginning and end of the study using the BIOPAC MP150/ECG BioNomadix system (sampling rate = 1000 Hz). Orthostatic recording conditions allowed us to minimize motion artifacts and measure trait HRV under rest. During each resting recording, participants were asked to sit in a comfortable position in a chair and try to refrain from moving while resting their gaze on a fixation cross (+) displayed in the center of the computer screen in front of them. No breathing instructions were provided because the normal vagal influence on the heart is best interpreted during spontaneous breathing (Berntson et al., 1997).

#### Social Reasoning Task

Participants were instructed via computerized instructions to “identify political/social issues relevant to Australians,” which stirred debate in the last few years and about which they felt strongly. Participants selected the societal issue from a list of pilot-tested topics of great relevance to Australians. These issues concerned education (e.g., funding cuts; 34.7%), environment (e.g., climate change; 15.3%), healthcare (e.g., hospital funding; 12.7%), unemployment (e.g., staffing cuts; 9.3%), economy (e.g., interest rates; 10%), politics (e.g., political divide; 8%), taxes (e.g., carbon tax; 4.7%), and social security (e.g., welfare; 5.3%). All selected topics were of high personal relevance (*M* = 4.15, *SD* = 0.79; i.e., above the scale midpoint), *t*_(149)_ = 17.80, *p* < 0.001.

#### Experimental Manipulation

We randomly assigned participants to the self-immersed or self-distanced conditions “to get your insight on factors that would be important to consider when imagining how this particular issue might unfold in the future.” In the *self-immersed* condition, participants were asked to “immerse themselves in the situation” and approach their thoughts from the first-person perspective. To facilitate this process, they were asked to “use the pronouns I/me as much as possible” as they tried to understand the thoughts they had. In the *self-distanced* condition, participants were asked to “focus on the situation” and approach their thoughts from a third-person perspective. Participants were instructed to use third-person (he/she) pronouns and their name as much as possible as they tried to understand the thoughts they had (Grossmann and Kross, 2014; Kross et al., 2014). The experimental manipulation was not repeated for the attribution judgment task.

In each condition, participants thought out loud about how the social issue they selected would unfold in the future, guided by the probes “How do you think this issue will unfold in the future? Why do you think the issue will unfold as you just described?” and their answers were audio-recorded (Grossmann et al., 2013).

#### Attributional Judgment Task

After a filler task, participants were invited to form “impressions about a stranger based on limited information about that person,” with the target person’s age and gender matched with that of the participant. Participants were presented with eight general descriptors (e.g., likes having fun with friends; dislikes too much work) and three specific cases depicting the person performing a neutral, positive, and negative act, with the latter two cases presented in a randomized order. Subsequently, participants’ spontaneous impressions of the person were recorded, followed by a second 6 min resting phase for physiological recording, and a medical history questionnaire.

### Measures and Data Processing

#### Heart Rate Variability

We trimmed the first/last 30 s to remove setting-related artifacts (e.g., posture adjustments by participants at the beginning or end of recording), and pre-processed the middle 5 min ECG recording sections using Kubios HRV 2.1 (Tarvainen et al., 2014). About 5% of participants showed significant posture adjustment-related artifacts in the beginning or end of recordings. For another 5%, the research assistant made an error in starting the recording late. So we chose to trim all recordings at the ends to extract the same length of 5 min recording. After identifying RR-time series via Kubios QRS-detection algorithm, a trained research assistant manually inspected recordings and corrected for missed artifacts within Kubios when necessary. Overall, the rate of false beats in the raw 5 min ECG recordings was low and varied from recording to recording (0–5% range).

Respiration was measured using a respiration belt and Biopac wireless BioNomadix module for concurrent ECG and respiration recordings. Respiration analyses were conducted using the Acqknowlege 4.2 Software. Each recording was also examined by a trained research assistant (who manually calculated a number of cycles per second) to double-check the software-aided respiration frequency calculation.

##### Selection of HRV indicators

There is a broad range of HRV indicators (Allen et al., 2007). Some of them capture the time-domain of the overall variability with the help of statistical parameters (e.g., standard deviation of the inter-beat interval [SDNN]; the root mean squared successive differences in the inter-beat interval [RMSSD]; the percentage of the absolute differences between consecutive inter-beat intervals that are greater than 50 ms [pnn50]). Others examine total variability based on the geometry of the RR histogram (e.g., the HRV triangular index obtained as the integral of the histogram divided by the height of the histogram [HRVTI] and the baseline width of the RR histogram evaluated through triangular interpolation [TINN]). Finally, some metrics assign bands of frequency and count the number of beat-to-beat intervals that match each band, e.g., by examining the fast fourier transform power spectrum density in the high-frequency band (HFPOWFF). Focusing on the high frequency is important given that empirically it is most closely aligned with the parasympathetic activity (Berntson et al., 1997).

Following recommendations for transparency and replicability in behavioral sciences (Simmons et al., 2011; Asendorpf et al., 2013), we sought to avoid cherry-picking of HRV markers yielding desirable results by examining a range of HRV markers (see Table 1). In line with prior research (e.g., Rainville et al., 2006; Allen et al., 2007), we also performed a principal component analysis (PCA) on HRV-markers, which yielded a one-component solution (variance explained: 88.88%; loadings: 0.91–0.97). To minimize the chance of α-inflation due to multiple testing, we performed primary statistical analyses on the retained standardized PCA factor scores.

**Table 1:** **Relationship between wisdom-related judgment (reasoning, balanced attributions) and heart-rate variability as a function of self-distanced vs. self-immersed condition**.

	Self-immersed condition			Self-distanced condition		
Type of HRV marker	Wise reasoning	Attrib. (a)	Attrib. (b)	Wise reasoning	Attrib. (a)	Attrib. (b)
SDNN	−0.001	0.06	0.09	0.25*	0.40***	0.36**
RMSSD	−0.08	0.09	0.12	0.26*	0.38***	0.37**
pnn50	−0.07	0.09	0.13	0.24*	0.40***	0.38***
HRVTI	0.01	0.03	0.07	0.23^†^	0.31**	0.28*
TINN	−0.02	0.03	0.08	0.21^†^	0.36**	0.33**
HFPOWFF	−0.09	−0.04	0.01	0.26*	0.41***	0.37**
Composite HRV index	−0.04	0.07	0.11	0.26*	0.38***	0.35**

Notes. We used partial Spearman’s correlations, controlling for bio-fitness indicators. HRV Index = score obtained from the loading on the 1st PCA component. Attrib. (a) = Balanced Attributional Judgment–3–level indicator. Attrib. (b) = Balanced Attributional Judgment—dichotomous indicator. ***p ≤ 0.001, **p ≤ 0.01, *p ≤ 0.05, ^†^p ≤ 0.11.

##### Trait vs. state-level variability

A single HRV measurement may have trait- and state-specific components. Our main interest is in the trait-level HRV. Hence, we focused on two resting measurement intervals, obtained before the manipulation, and after the attribution task. Averaging responses across several measurement points provides a more robust metric of an enduring trait-level individual difference (Mischel and Shoda, 1995; Robins et al., 2009). Preliminary analyses indicated that the HRV indicators did not significantly vary across time points. Further, experimental manipulation was not significantly related to HRV or respiration indices at either time point, *t*s < 1.40. Hence, in our main analyses we focused on the scores that we averaged across both measurement points (Bertsch et al., 2012), supplementing them with reports for each measurement point.

#### Wise Reasoning

Participants’ verbal reflections on the social issue were transcribed and coded by two hypothesis- and condition-blind coders on the following aspects of wisdom-related reasoning (Grossmann et al., 2010, 2013; Grossmann and Kross, 2014): recognition of limits of knowledge, recognition of possibility of change, consideration of others’ perspectives, and search for a way to integrate these perspectives/compromise; each coded on a 0 = “not at all” to 2 = “a great deal” scale (see Supplementary Appendix A for coding manual). Inter-coder reliability was good, as indicated by the intra-class correlation coefficients, ICC(2,1)_limits_ = 0.82; ICC(2,1)_change_ = 0.93; ICC(2,1)_perspectives_ = 0.95; ICC(2,1)_compromise_ = 0.87, with inconsistencies resolved via averaging.

Consistent with prior work (Grossmann et al., 2013; Grossmann and Kross, 2014), scores across different aspects of wisdom-related reasoning have converged on a single latent PCA factor (variance explained: 42.04%; loadings: limits = 0.83, perspective = 71, change = 0.66, compromise = 0.23). We retained the PCA scores for subsequent analyses. Due to a positive skew, scores (+min) were log-transformed for linear model analyses.

#### Attributional Judgment

The same hypothesis-/condition-blind group of coders counted spontaneous mentioning of disposition and situation when participants explained others’ behavior. Dispositional explanations were operationalized via statements referring to the person’s personality or moral virtues. Situational explanations were operationalized via statements referring to the situational forces influencing one’s behavior (see Supplementary Appendix B for coding instructions). Two participants did not follow instructions and were thus excluded from content analyses. Inter-rater agreement was acceptable, ICC(2,1)_disposition_ = 0.60, ICC(2,1)_situation_ = 0.61, with inconsistencies resolved via averaging. Preliminary analyses indicated a significantly greater proportion of attribution statements referring to personality (88%) rather than situation (38%), McNemar’s *χ^2^* (*df* = 3) = 83.36, *p* < 0.001, which is consistent with prior work (Ross, 1977). To quantify balanced (as compared to biased) attributional judgments, we examined whether individuals simultaneously considered dispositional and situational factors (32%). There are several ways to quantify balanced attributions. Therefore, we created two attribution indices to examine the generalizability of the results. First, we scored participants narratives from 1 = preference for one factor, but ignoring the other one, via 2 = no explicit mentioning of either factor, to 3 = simultaneous consideration of dispositional and situational factors. Second, we created a dichotomous index, ranging from 0 = biased attributions/no attributions to 1 = balanced attributional judgment.

#### Control Variables

We obtained a number of control variables, concerning mood, subjective importance of selected social issue, word length of the verbal reflections, and bio-medical history. Participants described their mood at baseline and after the attribution task (on a 1–7 scale; 1 = “extremely bad mood” to 7 = “extremely good mood”). Upon selecting the social issue, participants indicated how much they cared about the issue, and how important it was to them (on a 5-point scale; 1 = “not at all” to 5 = “very much”), averaged to create an index of personal relevance, *r* = 0.73, *p* < 0.001. To account for the essay length as a possible confound, we performed a word count of verbal reflections. Finally, participants reported exercise frequency in the past 3 months (1 = “0 h/week” to 4 = “over 10 h/week”; *M* = 2.27, *SD* = 0.86), weight and height (to calculate body-mass-index; *M* = 25.04, *SD* = 5.20).

#### Statistical Analyses

Analyses included Spearman’s correlations and multivariate general linear models for wise reasoning, as well as logistic regression analyses for the dichotomous index of balanced attributions. Because some of the variables were not on a continuous scale, we chose Spearman’s correlation as a conservative indicator of the magnitude of association.

## Results

Neither baseline mood, |*r*_s_s| < 0.07, nor post-reflection mood, |*r*_s_s| < 0.13, nor social issue topic, *F*s < 1.45 and *Wald* (*df* = 7) = 3.34, *p* = 0.85, significantly influence responses across variables of interest and will not be discussed further. Also, post-reflection mood was positively related to balanced attributions in the self-distanced condition, *r*_s_(3-level index) = 0.25, *p* = 0.03, *r*_s_(dichotomous index) = 0.24, *p* = 0.04, but not in the self-immersed condition, *r*_s_(3-level index) = −0.04, *r*_s_(dichotomous index) = 0.02. Participants with higher wisdom-related scores on the social issue task were somewhat more likely to spontaneously mention both personality and situation when judging others’ behavior, *r*_s_(both indices) = 0.14, *p* = 0.10.

**Figure 1:**
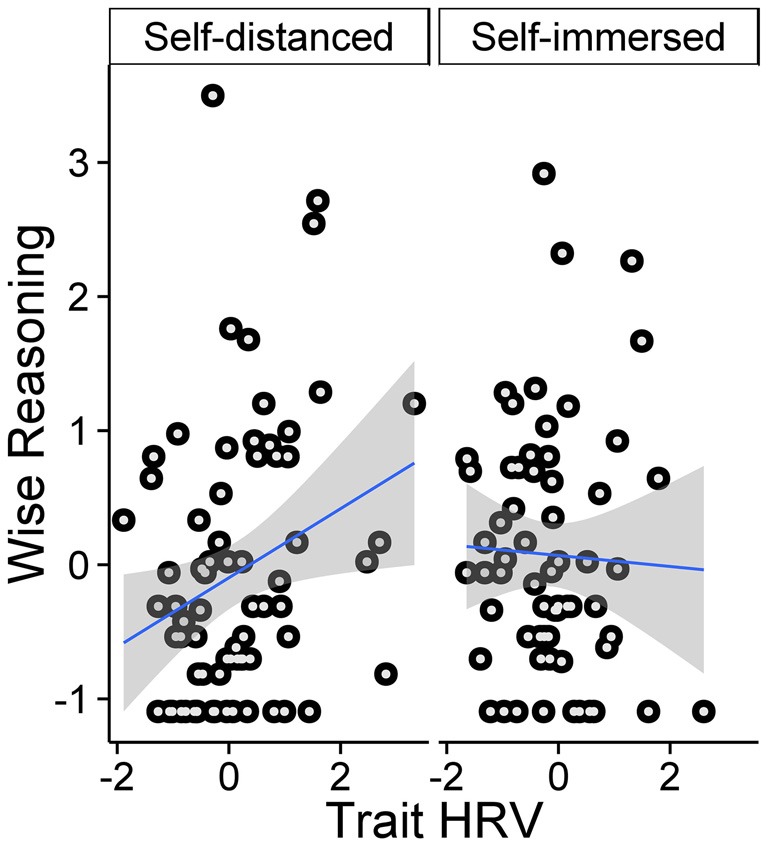
**Self-distancing moderates the relationship between heart rate variability (HRV) and wise reasoning (*z*-scores).** Scatterplot with line of best fit and 95% *CI*.

First, we examined how mean-centered condition (self-distanced vs. self-immersed), HRV-factor score, and their interaction predict wisdom-related reasoning. Bio-fitness metrics (age, BMI, exercise) and respiration rate were included as covariates; results without covariates were very similar. We observed no significant main effects, *t*s < 1, but, as expected, a significant condition × HRV interaction, *B* = 0.48, *SE* = 0.23, *t* = 2.07, *p* = 0.04, $\eta_{p}^{2}$ = 0.04. As Table 1 and Figure 1 indicate, in the self-distanced condition, each HRV-marker was positively associated with wiser reasoning. In contrast, there was no significant HRV-wisdom association in the self-immersed condition.

Next, we examined how mean-centered condition (self-distanced vs. self-immersed), HRV-factor score, and their interaction predict balanced attributional judgment (3-level index), performing identical analyses as above. Again, we did not observe a main effect of condition, *t* = 1.17, *ns*. We observed a significant positive main effect of HRV, *B* = 0.27, *SE* = 0.09, *t* = 3.02, *p* = 0.003, $\eta_{p}^{2}$ = 0.07, which was qualified by a marginally significant condition × HRV interaction, *B* = 0.33, *SE* = 0.17, *t* = 1.91, *p* = 0.059, $\eta_{p}^{2}$ = 0.03. As Table 1 indicates, in the self-distanced condition, each HRV-marker was positively associated with balanced attributions. In contrast, there was no significant HRV-wisdom association in the self-immersed condition.

**Figure 2:**
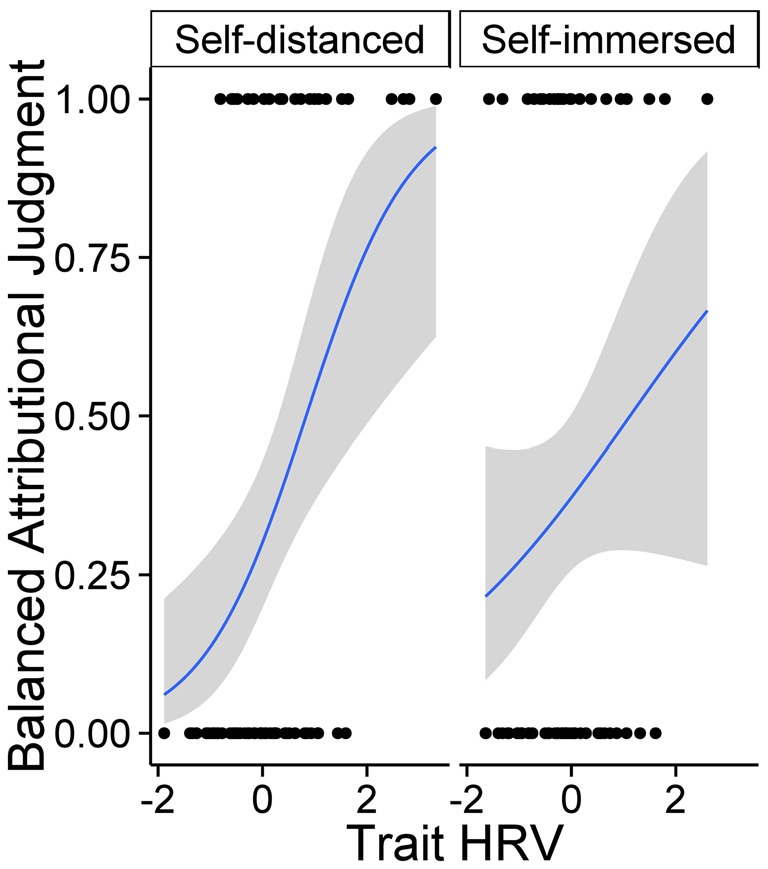
**Self-distancing moderates the relationship between HRV (*z*-score) and balanced attributional judgment (dichotomous index).** Log-likelihood estimates and 95% *CI*.

Finally, we performed comparable logistic regression analyses on dichotomous scores of balanced attribution. Again, results indicated a significant main effect of HRV, *B* = 0.75, *SE* = 0.24, *Wald* = 9.76, *p* = 0.002, *OR* = 2.12, suggesting that greater heart-rate-variability is associated with more balanced attributions. Moreover, as with a 3-level index of balanced attributions, this effect was qualified by a marginally significant condition × HRV interaction*, B* = 0.77, *SE* = 0.44, *Wald* = 3.06, *p* = 0.08, *OR* = 2.16. As Table 1 and Figure 2 indicate, HRV was positively associated with balanced attributions in the self-distanced, but not in the self-immersed condition.

The positive relationship between HRV and wisdom-related judgment in the self-distanced condition was robust when controlling for the length of verbal reflections, wise reasoning: *r* = 0.20, attributional judgment: *r*_s_(3-level index) = 0.35, *r*_s_(dichotomous index) = 32, and when separately examining each resting period used to obtain HRV indicators, 1st period: wise reasoning: *r* = 0.16; attributional judgment: *r*_s_(3-level index) = 0.35, *r*_s_(dichotomous index) = 31; 2nd period: wise reasoning: *r* = 0.29; attributional judgment: *r*_s_(3-level index) = 0.40, *r*_s_(dichotomous index) = 38. In light of prior work on the curvilinear effects of HRV on socio-emotional variables (Kogan et al., 2014), we performed additional analyses with HRV^2^. We did not observe any significant effects of HRV^2^, nor HRV^2^ × condition interactions on any of tested variables, *F*s < 1.

It is possible that the top performers on the wisdom-related judgment task could show higher HRV even in the self-immersed condition (see Figure 1). We tested this possibility in *post hoc* analyses, examining whether participants in the top 20% of the wise reasoning task performance showed higher HRV, and whether this effect was moderated by condition. Results of analyses with condition, wise reasoning performance group (top 20% vs. rest), and their interaction as predictors of the HRV, and bio-fitness metrics and respiration as covariates indicated a significant main effect of performance group, *F*_(1,117)_ = 6.69, *p* = 0.01, $\eta_{p}^{2}$ = 0.05, with the top performers (*M* = 0.50, *SD* = 1.04) showing greater heart-rate-variability than the rest of the sample (*M* = −0.09, *SD* = 1.04). Notably, the group effect was not moderated by condition, condition × group interaction, *F*_(1,117)_ = 0.31, *ns*.

## Discussion

The present work provides the first direct test of the relationship between HRV and wisdom-related judgment, demonstrating that the relationship was robust across both time-method- and frequency-method-based markers of high-frequency HRV. Further, HRV was linked to two distinct measures of wisdom-related judgment: one concerning participants’ spontaneous reasoning process about important social issues and another concerning balanced judgments of others’ behavior.

The central insight from the present research concerns the role of self-distancing for enabling high-HRV people to engage in cognitive processes philosophers and cognitive scientists have characterized as “wise”, namely those involving certain aspects of reasoning (e.g., intellectual humility, recognition of the world in flux and different perspectives on an issue) and evaluation of others in a less biased fashion. We introduced this article by pointing out that although we expect higher HRV-people to *able to* direct more resources to social information processing, we have no reason to expect that such processing will happen by default or be unbiased. Egocentric impulses (Foster et al., 2003) may guide people towards the self-serving features of the social issue at hand, leading them to neglect the non-self-serving features, including interests and opinions of other individuals involved in the issue. As a consequence, in the absence of a cue to self-distance, high-HRV people may be similarly prone to a bias in judgment as their low-HRV counterparts. Further, we proposed self-distancing would aid high HRV people to curb egocentric impulses and utilize their cognitive resources for a judgment that is not only efficient but also wise. Our results were consistent with this proposition. Whereas there was no relationship between trait-HRV and wisdom-related judgment in the self-immersed perspective, adopting a self-distanced perspective led to a positive association between trait-HRV and wisdom-related judgment.

A *post hoc* analysis comparing the top 20% of the wise reasoning distribution to the rest of the sample revealed a notable exception to this observation. Top performers on wise reasoning indicated a significantly higher cardiac vagal tone than the remainder of the sample, irrespective of the condition these participants were in. This effect was true for both self-immersed and self-distanced participants. Though tentative, it is possible that the top 20% performers on wise reasoning show habitually high level of cardiac vagal tone, in contrast to the rest of the population that benefits from self-distancing to channel their vagal tone for a wise judgment. Another possibility is that the top performers on wise reasoning are more likely to *spontaneously* transcend their immediate viewpoint, thus aligning their cardiac vagal tone with wisdom-related cognitions. Future work can explore these possibilities by examining wise reasoning and HRV-related psychophysiology across multiple situations. Such multi-situation assessment would allow to disentangle state- from trait-level processes (Mischel and Shoda, 1995; Fleeson and Noftle, 2008), as well as assess the role of situational contingencies for wise thought. For instance, a multi-session design will allow testing whether people who are high in cardiac vagal tone are equally wise in the domains of friendship, politics, and love.

These results unite and extend several streams of research. The observed moderation between trait-HRV and wisdom-related judgment is consistent with prior observations about the conditions under which traits align with one’s thoughts and behavior. Past research indicates that psychological distance promotes greater consistency between one’s core self-characteristics and one intentions (Wakslak et al., 2008), and between one’s intentions and behavior (Ledgerwood et al., 2010). The present study extends this body of research, for the first time testing how psychological self-distancing and trait-level physiology interact to influence wisdom-related judgment.

The present work also extends prior research in cardiac psychophysiology, further exploring the relationship between HRV and prosociality (Keltner et al., 2014). We observed a positive relationship between higher HRV and wisdom-related judgment when instructing participants to adopt a self-distanced perspective. This observation dovetails with other psychophysiological work showing a stronger positive relationship between HRV and one’s relational well-being when practicing loving-kindness meditation (Kok et al., 2013), which consists of key self-distancing components.

In contrast to prior research (Kross and Grossmann, 2012; Grossmann and Kross, 2014), the present study found limited evidence of self-distancing impacting wise reasoning directly. There are several notable differences between the extant prior research and the present study. First, prior research has focused on personal dilemma (Kross and Grossmann, 2012; Grossmann and Kross, 2014) while the present study concerned reasoning about political issues pre-selected to be of general relevance to Australian population. In line with prior theorizing (Grossmann and Kross, 2014), it is possible that self-distancing is a more potent determinant of wiser judgment when the issue concerns one directly. Second, there are important procedural differences between present and prior experiments. Kross and Grossmann (2012) employed visual and spatial manipulations of self-distancing, whereas the current study has employed the 3rd- vs. 1st-person self-talk manipulation of self-distancing (Kross et al., 2014). Further, whereas (Grossmann and Kross (2014); Studies 2–3) have employed a similar self-talk manipulation as the one in the present work, the focus of their studies concerned reflecting on interpersonal transgressions, whereas the present study concerned *prospecting*—i.e., thinking about future developments of a political issue. Past research has shown that prospection is distinct from reflection on such parameters as level of abstraction (Trope and Liberman, 2010), as well as the role of memory and affective processes (Seligman et al., 2013). It is possible that the self-talk manipulation of self-distancing is more potent at boosting wisdom about concrete situations concerning the individual reflecting on the issue, as compared to prospecting about future events of general societal relevance.

How does self-distancing promote greater wisdom among high HRV individuals? Based on the neurovisceral integration model linking higher heart-rate-variability to the functioning of the executive circuits in the brain (Thayer and Lane, 2000; Thayer et al., 2012), we proposed that self-distancing instructions prompt high-HRV people to utilize their executive abilities in non-egocentric fashion, resulting in wiser judgments. Future work is needed to test the exact mechanisms through which self-distancing could facilitate utilization of executive circuits towards wise judgment and behavior. Research is also needed to examine whether increasing HRV, especially amongst low HRV participants, increases sensitivity to self-distancing cues. If a person’s heart were to become more variable and “flexible,” for example through meditation practices (Krygier et al., 2013), would this result in the person being more able to self-distance and reason wisely?

## Conclusion

Though wisdom has been long viewed as too ethereal to be a subject of a tangible empirical inquiry, in the last 25 years researchers have established several psychological components of wise judgment (Staudinger and Glück, 2011). Recently, neuroscientists have proposed that to understand individual differences in wisdom one also ought to consider aspects of human physiology (Meeks and Jeste, 2009). The present article does exactly that, focusing on HRV. Our research suggests that wisdom-related judgment is not exclusively a function of the body or the mind. Rather, both greater heart-rate-variability and an ego-decentered mind are required for a wiser, less biased judgment.

## Author Contributions

IG and BKS designed research. JC provided valuable feedback and modification suggestions for study design. BKS conducted research. BKS analyzed psychophysiological data. IG supervised wisdom- and attribution-related content-analyses of the participants’ narratives. IG performed statistical analyses and drafted the first draft of the manuscript. BKS and JC provided valuable feedback on the manuscript. All authors approved the final version of the manuscript for publication.

## Funding

The present research was funded by Social Sciences and Humanities Research Council of Canada Insight Grant 435-2014-0685 (to IG) and by a grant from the John Templeton Foundation, “Prospective Psychology Stage 2: A Research Competition to Martin Seligman” (sub-grant awarded to IG), the Mind and Life Contemplative Studies Fellowship (to BKS), and a grant from the Australian Research Council (to JC). The opinions expressed in this publication are those of the author(s) and do not necessarily reflect the views of the John Templeton Foundation.

## Conflict of Interest Statement

The authors declare that the research was conducted in the absence of any commercial or financial relationships that could be construed as a potential conflict of interest.
