# Supplementary material for: A Heart and A Mind: Self-distancing Facilitates the Association Between Heart Rate Variability, and Wise Reasoning
Source: Front Behav Neurosci. 2016 Apr 8;10:68. doi: 10.3389/fnbeh.2016.00068 (PMC4824766; doi:10.3389/fnbeh.2016.00068)
Supplement: Supplementary file 1 [file DataSheet_1.docx]

Supplementary Material

A Heart and a Mind: Self-distancing Facilitates the Association between Heart Rate Variability, and Wise Reasoning

Igor Grossmann1*, Baljinder K. Sahdra2, Joseph Ciarrochi2

1Department of Psychology, University of Waterloo, Waterloo, Ontario, Canada

2Institute for Positive Psychology and Education, Faculty of Health Sciences, Australian Catholic University, Strathfield, New South Wales, Australia

* **Correspondence**: Igor Grossmann: igrossma@uwaterloo.ca

# Supplementary Appendix

**Part A: Coding Guide for Aspects of Wisdom-related Reasoning**

**First, we rule out people who answer all questions with “I don’t know”. These participants will not be included in further analyses.**

**Recognition of limits of knowledge**

**(0 = none, 1 = some, 2 = a great deal)**

*This dimension deals with recognizing that one’s knowledge on the subject matter is limited, that they need more information. Pointing out that one’s knowledge/opinion is contextual and knowledge/opinion of someone else may be different from one’s own also belongs to this category. Note: there may be some overlap between the concept of limits of knowledge and consideration of others’ perspectives. That is expected, as these two categories mutually influence each other.*

Utterances scored HIGH on recognition of limits of knowledge could address questions like this:

Do we know everything that there currently is to know? Is there a possibility that there are things that we don’t know? Do we look for the opinions of others to inform our solution, without assuming that we know what others know?

**0: No recognition of limits of knowledge**

*This country is going to fall to the ground*. All employment is going to go overseas, and Australia is going to be left with *nothing*. (12-1)

| Subject does not recognize that his/her knowledge is limited. This response also indicates no recognition of uncertainty as he/she assumes that this is the only way this issue can and will unfold. |
| --- |

Well I’m pretty sure because Alex studied about it, I’m kind of studying in university, I’m doing my degree, so we’ve been taught about it (40-2)

| Subject fails to recognize limit of knowledge as he doesn’t realize what he studied situated in a given context, and thus could be biased, context-specific or simply wrong. In response to “why do you think the issue will unfold as described,” subject indicates it will unfold as described simply because he has been taught so. |
| --- |

**1: Has some recognition**

Stephanie thinks that *depending on the power* on the political power, *it doesn’t look very good* for the future. (21-1)

| Subject suggests some relativism of the issue - the future is dependent on what political party comes into power. However, subject does not elaborate on this issue further, making a generally negative forecast. |
| --- |

Um, I think *it’ll depend on the election* this year. But *regardless, I think that* the cost of education will be cut and that will impact how poor people would be able to move into a better position economically and socially (22-1)

| Again some recognition that the future is context-dependent. However, subject throws in his or her own opinion as to how the future will unfold, irrespective of the consideration of limited knowledge. |
| --- |

**2: Fully recognizes limits of knowledge**

*Now being such a large issue, I can’t say at right* that taxes should be increased across the border. But when the decision to increase or decrease taxes should be purely empirical and not based on culture or how everyone feels about it, it should be a pure numbers game and supported by actual experts (4-3)

| Subject realizes the future of the issue is context-dependent, and that how the future will unfold is not up to him or her. Subject also realizes that the discussion of the issue should not be based on a single person’s or the public’s opinion, that public knowledge is limited and that only actual experts (who are knowledgeable about the issue) could accurately predict how the future will unfold. |
| --- |

I think Megan should *spend some time reflecting more* on the issue and find out where the gaps are in what she thinks she needs to know and *looks at some resources* that are around her, and that she could educate herself a little bit better by *talking* *to* *some* *people* who are more familiar with the topic, and perhaps do some *workshops* around, cultural issues, and *look at some of the* *better* *press* on the topic, and gain some competence in the area. (7-3)

| Subject recognizes that her knowledge is inadequate and limited. Subject suggests doing research as ways of educating herself to become more familiar with the topic. Subjects also suggest a variety of ways in which she could gain information, such as workshops, talking to experts, reading cultural issues and press coverage etc. This response also indicates a high level of perspective-taking, because this subject seeks to consult many different perspectives about the issue. |
| --- |

**Recognition of possibility of change (in current system)**

**(0 = none, 1 = some, 2 = a great deal)**

*The key idea here deals with change of the direction of the system. A system can refer to the relationship between discussed concepts in motion (e.g., “the attitude to this issue is getting worse and worse”). The key criterion for high scores on this dimension is .awareness of possibility that the trajectory may change (instead of a linear continuation).*

Note, “improvement” does not necessarily imply change. To assess change, you must first establish the current direction of change (i.e. do not focus on how things have been in the past, but how things are in the present), and compare that participant’s perceived future trajectory. E.g., if things are better now than they have been in the past, and subject predicts that things will continue to improve in the future, it is a 0.

To be able to qualify as a 1, participant must acknowledge that the future is either a) unpredictable or b) able to unfold neutrally or positively and negatively (e.g. “it looks like it’s getting worse, but you never know”).

Change is different from what should be done, it is what the subject thinks will be done.

**0: No recognition of possibility of change**

Because that's the way I see it in the moment, and it doesn't seem to be changing. (1-2)

He thinks the issue will get worse and worse because politicians keep making bad decisions without much thought as to the people they’re actually going to affect. (13-1)

Lauren thinks that taxes are only gonna get worse, as time goes on and people are paying for things that aren’t really what they’re working for. (85-1)

| Subjects in these responses above indicate that the current state of the system is undesirable and that consequences will unfold as they described because of this state. Their views suggest that the future is bleak and they do not realize that things could potentially change in the future. |
| --- |

**1: Some recognition of possibility of change**

Decara thinks that the current scheme that’s trying to be put in place with *Gonski probably isn’t going to work*, rating each school only on the basis by its grades and rating each teacher by the grades that are produced within that classroom is sort of counter-productive to what education is about. (17-1)

| The above response suggest that there may be a slight possibility of change, or that something about the current system may not work out. Subject does not elaborate on the nature of change, it is merely implied in the italicised part. Hence, this subject does not score as “full recognition of change.” |
| --- |

Hopefully they’ll be able to see the consequences as to why they made this decision for universities and hopefully they’ll be able to use like a different source of funding instead of university next time because pretty much the main source of using high schools is to get into higher education such as universities and employment and so on. (75-2)

| The subject is “hopeful” that the authorities will realize some time in the future that change is needed. Because the nature of change is vague and only implied, this utterance does not receive full change consideration. |
| --- |

**2: Fully recognizes the possibility of change**

I think the old style of political parties, where we just got 2 political parties, *I think it’s gonna change*. I think people are really sick of big party politics, *and I think there will be more smaller groups coming into the political arena*. And I could believe that *one day the government comes about by a collection of group rather than just one party* coming and do whatever they like for 3 or 4 years ... one of the disadvantages we’ve got in politics in Australia at the moment is that everything seems to be with a one year or two year or three year plan, *I think we need a ten year plan and I don’t think we plan well for the future*. I think we just lurk from one situation to the next I don’t think that’s good planning … (30-1)

I think that *a lot of things are changing,* specifically due to the political climate of today, specifically of the last couple of weeks. (35-1)

| In the above responses, subjects recognize and acknowledge the possibility of change – that even though things are not working out at the moment, things could change in the future, or that the system is constantly being modified so the future could be uncertain. |
| --- |

**Integration of different perspectives / Search for compromise**

**(0 = none, 1 = some, 2 = a great deal)**

*Searches for a fair agreement, a middle-ground.*

Note, there is a distinction between equality and compromise: Sometimes, when people ask for equality, they're asking for the current system to have the more privileged compromise and the poor to just accept what the rich can give back to them (in this logic, the less privileged are not really "compromising" since they have nothing to compromise).

Also, “most positive for all” does not necessarily imply compromise without an indication unless a person really talks about both parties giving up something (or hints in that direction).

Suggesting compromise in a way that promotes only one side of the argument should not be considered compromise (look at the response as a whole).

On the other hand, an individual compromise (i.e. a compromise in one’s own interest) can also be considered. E.g., to sacrifice current ways of life for future sustainability (the individual must be included in the compromise, the compromise should not be prescribing actions for “everyone else”).

**0: No research for compromise**

*The Australia government should keep all work in Australia instead of sending manufacturing overseas*, giving Australia less taxes, pay our people, instead of looking after other country’s people.

| Subject does not seem to recognize that there are opposing perspectives and s/he is not interested in figuring out a way to work things out between these perspectives Thus there is no search for an agreement, or any sort of compromise. |
| --- |

**1: Some search for compromise**

N/A (because no-one has yet given a mid-level answer). Ideally, this statement would indicate clear indication of opposing perspectives, an indication of acceptance of these opposing perspectives, without integration of these perspectives in a search for a compromise between them.

**2: A great deal of search for compromise**

*There should be some kind of way to regulate everything so that people do get similar pay* but then I suppose it would make it more difficult for people who have immigrated to gain work but um yeah I don’t know what the answer is but *there should be some kind of way to make it a little bit more fair* ‘cause at the moment it doesn’t seem good for either side but how that should be solved I don’t have a good answer for that. (16-3)

| Subject suggests a fair agreement. The subject indicates a clear search for compromise that would benefit both sides concerned by the issue. |
| --- |

**Perspectives taking**

**(0 = none, 1 = some, 2 = a great deal)**

*Consider the perspectives of different people involved (e.g., jobs are difficult to find for BOTH domestic and international students)*

Note, participants can perspective-take by appealing to or pointing out the opinions of others. Two key factors that determine whether or not a participant is high in perspective-taking are his/her attitudes when perspective taking (e.g. negative, neutral or positive) and the breadth of perspective-taking (e.g. are they trying to get at the issue from every possible angle). When perspective-taking attitude is negative (e.g., discounting without elaboration), it doesn’t not deserve a high score on this dimension. Regarding the breadth of perspective-taking, the participant must acknowledge a perspective different from their own.

**0: No perspective taking**

To begin with, *private schools should not be given as much funding and public schools should be given more because if they go to a private school their parents obviously have enough money to send them there*…(19-3)

*The Australian government doesn’t care about Australian people*. They ‘re sending money overseas, sending jobs overseas, sending work overseas and Australians are left without work, can’t support their family. (12-2)

| Subjects in the above responses are not interested in considering the perspectives of other people who may not necessarily share the same interest as them. |
| --- |

**1: Some perspective taking**

In terms of governments trying to um make quick fixes for education, *they really should be talking to academics, rather than just making policies that will buy them votes*. (3-3)

I think that the Australian government should *look at the level of education in other countries* such as European and Asian, perhaps *take on the perspectives that they take,* they should look at statistics within these countries and in Australia and see how the children education in other countries compare... (5-3)

| The above responses indicate some willingness to perspective-take, but the subjects are only willing to consider the perspectives of certain, yet not all, people involved, hence these subjects meet criteria for some perspective taking and not full perspective taking. |
| --- |

**2: Full perspective taking**

I understand that people need the jobs but from *both sides* whether it’s people with citizenships or people who immigrated here they’re obviously looking for it for a reason. (16-3)

Russell thinks that we should really *research* how much education is helping us, whether it’s comparing that to other countries, or racially look at the population at other areas that aren’t as educated... (28-3)

| Subjects consider the different perspectives of different parties involved. |
| --- |

**Part B: Coding Guide for Dispositional and Situational Attributions**

**Dispositional & situational attributions of protagonist’s (Alex’) action**

**(0 – no mentioning vs. 1 - mentioning)**

**Dispositional Attributions**: Participant explains protagonist’s behavior by indicating it’s “how Alex is,” referring to his/her personality, moral virtues, etc. (dispositional attributes). Mention of “personality” factors: Alex took X action because he is Y/Z (personality factor) (If yes – 1; if no - 0).

**Situational Attributions**: Participant explains Alex’s behavior by considering the situational forces that might have make Alex perform the positive or the negative act . Mention of situational factors: Alex took X action because of the situation (If yes – 1; if no - 0). Explains behaviour in terms of the specific situation.

If not mentioned, the rating for either is (0). Explaining that the situation happened is not situational or dispositional, must explain why Alex did it (referencing situation or personality).

Note: Using words like “Alex seems normal, average, or okay” does not count as an attribution.

***Dispositional Attributions***

Alex is a douchebag. My impression is based mainly on the whole pizza scenario, I just find that wrong. (67-4)

The fact that he’s not willing to engage with others on issues of politics or educational be it whatever, either shows he’s a very introverted person or very unintelligent person. (6-4)

| In the above responses, subjects attribute the reasons for Alex’s behaviors to his personality, something that is dispositional and cannot be changed (e.g., he did this so he must be a bad person), with little considerations given to situational forces (e.g., perhaps Alex was rude because he was having a bad day). |
| --- |

***Situational Attributions***

In the ride scenario, hey it’s public transport, I don’t think I’d be really interested in talking to a complete stranger about an issue, so don’t blame them. (46-4)

I thought that the first scenario when Alex was on the train she might’ve been a little bit rude but it was maybe because she had a long day and didn’t wanna stand up on the train. (59-4)

Ordering pizza on a friend’s credit card and not telling them about it but depending on if she knew the friend or anything like that it might’ve been a little bit of a joke or something that they wouldn’t minded. (59-4)

| In the above responses, subjects take into consideration the situational forces that might have influenced Alex’s behaviors. For instance, Alex was tired from a full day of work, or that nobody would want to talk to a complete stranger on a crowded train. The last response considers an implicit norm that might have existed in between Alex and his roommate instead of just simply concluding that Alex is a bad person because of his unpleasant behaviour. |
| --- |
